# Role of Sodium Dodecyl Sulfate in Tailoring the Rheological Properties of High-Strength Gelatin Hydrogels

**DOI:** 10.3390/gels7040271

**Published:** 2021-12-16

**Authors:** Virginia Martin Torrejon, Yanqiu Deng, Guidong Luo, Bingjie Wu, Jim Song, Song Hang, Dongmei Wang

**Affiliations:** 1 Media and Communication School, Shenzhen Polytechnic, Shenzhen 518055, China; virginia2020@sustech.edu.cn; 2 Department of Applied Chemistry, School of Science, Xi’an Jiaotong University, Xi’an 710049, China; 3 School of Innovation and Entrepreneurship, Southern University of Science and Technology, Shenzhen 518055, China; 12032573@mail.sustech.edu.cn (Y.D.); 12032564@mail.sustech.edu.cn (G.L.); 12032551@mail.sustech.edu.cn (B.W.); songjh@sustech.edu.cn (J.S.); songh3@sutech.edu.cn (S.H.)

**Keywords:** gelatin–SDS hydrogels, rheology of gelatin hydrogels, gelatin gelation kinetics, melting and gelling temperature of gelatin gels, gelatin–surfactant interactions

## Abstract

Gelatin hydrogels are widely used materials that may require surfactants to adjust their solution’s surface tension for cell attachment, surface adsorption enhancement, or foaming. However, gelatin is a highly surface-active polymer, and its concentrated solutions usually do not require surfactants to achieve low surface tension. However, anionic surfactants, such as sodium dodecyl sulfate (SDS), interact strongly with gelatin to form complexes that impact its hydrogels’ rheological properties, influencing processability and functionality. Nevertheless, there is a lack of systematic research on the impact of these complexes on high gelatin content (i.e., high strength) hydrogels’ rheological properties. In this work, the SDS/gelatin ratio-dependent viscoelastic properties (e.g., gel strength, gelation kinetics, and melting/gelling temperature) of high-strength gelatin hydrogels were investigated using rheology and correlated to surface tension, viscometry, FTIR, and UV-Vis spectrophotometry. SDS–gelatin ratio was proved to be an important factor in tailoring the rheological properties of gelatin hydrogels. The gel strength, gelation kinetics, and melting/gelling temperature of the gelatin hydrogels linearly increased with SDS incorporation up to a maximum value, from which they started to decline. The findings of this work have wide applicability in tailoring the properties of gelatin–SDS solutions and hydrogels during their processing.

## 1. Introduction

Hydrogels from natural polymers have attracted research and development attention for a wide range of applications due to their biodegradability, biocompatibility, and ability to mimic living tissues’ characteristics [1,2,3]. Among biopolymer-derived hydrogels, gelatin is considered one of the most promising due to its thermoreversible gelling properties, film-forming ability, and processing versatility [4,5,6]. However, it is important to tailor their mechanical strength, rheological properties, and thermal stability to meet their target functional and processing requirements [7,8,9]. These can be achieved by formulation (e.g., gelatin concentration and incorporation of surfactants, crosslinkers, nanomaterials, or polymers) and the selection of processing methods and conditions (e.g., thermally induced phase separation, foaming, 3D printing, electrospinning, and porogen leaching) which can alter the gelatin hydrogels’ molecular structure and properties [7,8,9,10,11].

Surfactants can alter gelatin hydrogels’ properties due to protein reconfiguration and complex formation caused by gelatin–surfactant interactions. These complexes are usually visualized as surfactant micelles bound into the gelatin’s polypeptide chains like beads in a necklace [12,13,14,15,16]. Surfactants are used to modify gelatin solutions’ surface tension for cell attachment, surface adsorption enhancement, foamability, and stability of emulsions in different applications, including wound dressing [10], additives encapsulation [17,18], and cosmetics [19]. As surfactant concentration increases in aqueous solutions, the surface tension usually decreases until reaching the critical micelle concentration (CMC). This is the concentration at which the surface adsorption of a surfactant at the liquid/gas interface saturates, and surfactant molecules start to aggregate into micelles by electrostatic forces [20]. However, gelatin is highly surface-active, and in concentrated solutions, it competes with surfactants for surface absorption. Thus, surfactants are not usually suitable for further decreasing the surface tension in concentrated gelatin solutions [21].

The formation of gelatin–surfactant complexes can also alter gelatin hydrogels’ rheological properties, such as gelation kinetics [22,23,24,25] and gel strength [21,26,27], which, in turn, affect their processability and applicability. For example, a hydrogel’s rigidity must be enough to sustain itself and adhere to a target tissue or stabilize liquid foams, while proper flow properties are necessary for production methods such as foaming, casting, or extrusion.

The gelation of gelatin hydrogels arises from the transition of its polypeptide chains from a coiled state when dissolved in warm water to a renatured triple helix configuration on cooling. During gelation, the water is trapped within the gelatin polymer chains, and a more rigid structure is held together by combining intermolecular forces, such as hydrogen bonds, electrostatic forces, van der Waals forces, and hydrophobic interaction [20]. Gelatin solutions transform from a near Newtonian liquid to a viscoelastic hydrogel, and the gel strength is reflected by the increase in the storage modulus *G’*, which is related to the amount of triple helix present in the hydrogel structure. Gel strength depends on several factors, such as temperature, time, and gelatin type and content. A strong relationship has been found between the storage modulus and surfactant concentration in gelatin-based hydrogels. The elasticity and gelation kinetics of gelatin–surfactant systems were reported to increase with surfactant concentration up to a maximum value from which they started to decrease, leading to protein denaturation and softening [28,29].

Sodium dodecyl sulfate (SDS) is a widely used anionic surfactant that has been used to modify hydrogels’ properties for different applications [23,30,31,32]. Anionic surfactants, such as SDS, strongly interact with gelatin, a polyampholyte with positive and negative charges distributed along its molecular backbone [33]. Thus, there is a considerable practical and theoretical interest in studying the effects of anionic surfactants on gelatin hydrogels’ properties. In addition, it is crucial to understand gelatin–surfactant interactions and their impact on viscoelastic properties to provide formulation guidance for novel gelatin processing methods and applications that require high-strength hydrogels. However, while many studies shed some light on the mechanism of gelatin–surfactant interactions, the focus has been mainly on viscosity [13,32,33], surface tension [24,34,35] and rheology in low-strength gelatin hydrogels [24,29,36]. Thus, relatively little work has been done on the systematic rheological characterization of high bloom and concentrated gelatin hydrogels with a wide range of gelatin and SDS concentrations [21,27,29,36]. There is also a lack of research on the temperature-dependent rheological properties of gelatin–SDS high-strength hydrogels, which are essential in controlling hydrogel processing (e.g., foaming, electrospinning, or 3D printing).

Therefore, this study aimed to investigate the extent to which SDS influences the rheological properties (gel kinetics, gel elasticity, and temperature-dependent behavior) of high-strength gelatin–SDS hydrogels in order to gain a better understanding of gelatin–surfactant interactions and their influence on the optimization of gelatin hydrogels’ rheological properties. The contributions made here have wide applicability on the processing of gelatin-based hydrogels and solutions. While SDS did not significantly modify the gelatin solutions’ surface tension, it considerably influenced their rheological properties. Lower SDS concentrations resulted in an increase in rheological properties, while excessive SDS concentrations caused their decline.

A systematic small-amplitude oscillatory shear (SAOS) rheological study covering a wide range of gelatin and SDS concentrations was carried out in correlation with the characterization of surface tension, viscometry, and FTIR.

## 2. Results and Discussion

### 2.1. Complexes’ Solubility

**Figure 1:**
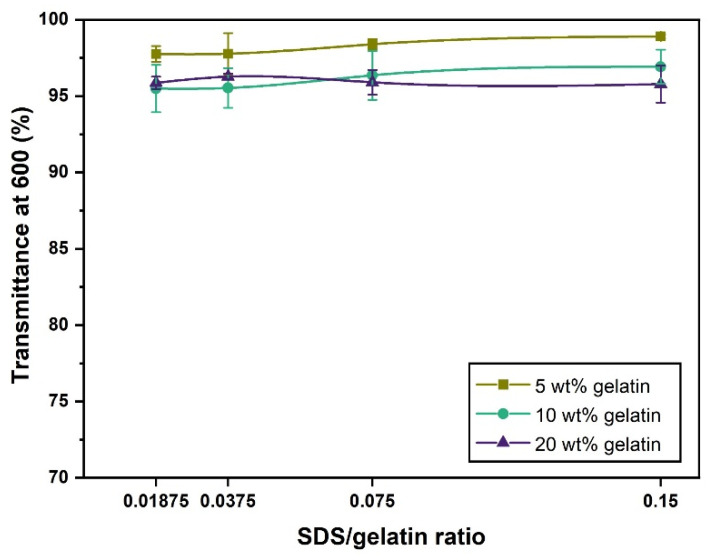
Transmittance (%) at 600 nm as a function of S/G ratio for 5, 10, and 20 wt.% hydrogels at their natural pH and 40 °C.

Polymer–surfactant interactions can alter the solubility of polymer solutions [37]. A decrease in the solubility of the complex can lead to certain issues, such as precipitation, fabrication hindrance, and deceptive rheological results. More precisely, ampholytic hydrogels and anionic surfactant associations can lead to changes in the protein topology and conformation, but their interactions usually lead to fully solubilized and relatively stable complexes [38,39]. UV-Vis spectrophotometry results confirmed the solubility of the gelatin–SDS complexes at their natural pH and the temperature at which the characterization was performed. As seen in Figure 1, the gelatin–SDS solutions’ transmittance values at 600 nm were higher than 95% in the studied range of S/G ratios and the three gelatin concentrations (5, 10, and 20 wt.%). This implies that the gelatin–SDS complexes were soluble at their natural pH and 40 °C, the conditions in which the rheological study was carried out. Higher gelatin concentrations tended to exhibit slightly lower transmittance values as the solution became more concentrated. On the contrary, higher S/G ratios tended to depict a slightly higher transmittance, attributed to protein denaturation and complex solubilization at higher SDS concentrations [40].

### 2.2. Surface Tension

**Figure 2:**
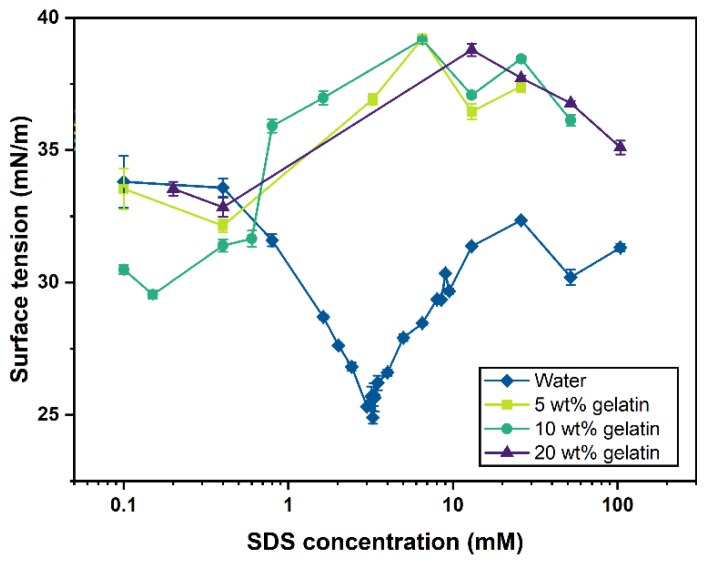
Surface tension vs. surfactant concentration (log) relationship in water and gelatin solutions at 5 wt.%, 10 wt.%, and 20 wt.% gelatin content at 40 °C.

Gelatin can adsorb and associate with SDS by electrostatic and hydrophobic forces [37,41]. At low surfactant concentrations, this association is mainly driven by gelatin–SDS monomer interaction, while when above CAC (critical aggregation concentration), SDS micelles’ formation also leads to the formation of gelatin–micelle complexes. Figure 2 displays the surface tension of the SDS aqueous solutions (without gelatin) and gelatin–SDS solutions at 5, 10, and 20 wt.% gelatin concentrations at their natural pH and 40 °C temperature.

The surface tension of the deionized water at 40 °C was 69.74 ± 0.34 mN/m, in agreement with the value of 69.6 mN/m reported by the Engineering Toolbox [42] at the same temperature. As SDS was incorporated into deionized water, the surface tension decreased to a minimum value at the so-called CMC, where surfactant monomers cover the air-water interface and start to associate via their hydrophobic tails to form micelles. Due to temperature influence, the CMC of the SDS aqueous solution at 40 °C identified here was 3.26 mM, lower, as expected, than the value of about 8–10 mM commonly reported in the literature for solutions in the absence of any electrolyte at 25 °C [12,43]. Beyond CMC, the surface tension of the SDS aqueous solutions was generally expected to exhibit a relatively constant value. However, Figure 2 exhibits a slight increase that may be attributable to the presence of impurities in the SDS [43].

Pure gelatin solutions in the absence of SDS were highly surface active. Compared to that of water, the surface tension of the pure gelatin solutions decreased by around 50% to 35.8, 35.2, and 36.2 mN/m for the 5 wt.%, 10 wt.%, and 20 wt.% gelatin solutions, respectively. In addition, there was a slight further reduction of surface tension (CAC) in the gelatin–SDS solutions at low SDS concentrations (<0.3 mM). The low value of CAC compared with that of the CMC for SDS aqueous solution indicates the competitive absorption of gelatin and SDS at the solution/air interface, resulting in premature saturation at the interface. Furthermore, the fact that the surface tension of the gelatin–SDS solutions was slightly higher than that of the pure SDS aqueous solutions provided additional evidence about gelatin and surfactant absorption competition at the solution/air interface.

When the surfactant concentration was above the CAC, the surface tension increased (around 10 mM) to a level around 39 mN/m for all three gelatin concentrations. Thus, the gelatin–SDS system had a slightly higher surface tension than the pure SDS solutions at the studied SDS concentrations, from which it can be inferred that gelatin–SDS complexes are less surface-active than SDS. Consequently, the SDS addition for the benefit of surface tension reduction was largely compromised. As surfactant concentration increases beyond the values studied in this work, it has been shown that gelatin is desorbed from the surface, leaving just a monolayer of surfactant at the interface and leading to surface tension values similar to those in the SDS–water solutions [18,37].

### 2.3. FTIR Spectroscopy

FTIR spectroscopy was used to provide information about the secondary structure adopted by the polypeptide chain of 5 wt.% and 20 wt.% in gelatin and gelatin–SDS dry gels. The main infrared absorption bands for proteins in the IR spectra are situated around 3300–3350 cm^−1^ (amide-A region), 3100 cm^−1^ (amide-B), 1600–1700 cm^−1^ (amide-I), and 1550 cm^−1^ (amide-II). Amide-I, especially, and amide-II are the most closely related vibration bands to the secondary structural elements of the protein backbone [44]. Amide-I almost entirely represents the C=O stretching vibration of the peptide bond (80% of the potential energy) and, to a lesser extent, in-plane N–H bending (<20%) [44], both involved in the hydrogen bonding taking place in the development of the secondary structure of proteins. The amide-II band shows less protein conformational sensitivity than the amide-I band as it mainly arises from the N–H groups bending (40–60%) and C–N stretching vibrations (18–40%) [44]. Amide-A represents N–H stretching and hydrogen bonding, while amide-B mainly depicts N–H stretching vibrations [45].

**Figure 3:**
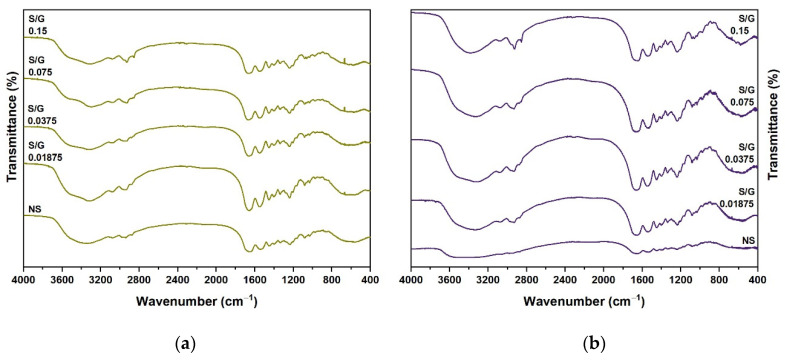
(**a**) FTIR spectrum of 5 wt.% gelatin dry gels and 5 wt.% gelatin gels at different S/G ratios. (**b**) FTIR spectrum of 20 wt.% gelatin dry gels and 20 wt.% gelatin gels at different S/G ratios.

Table 1 shows the frequency values of the main infrared absorption bands, while Figure 3 compares the intensities of the different peaks. The 5 and 20 wt.% gelatin and gelatin–SDS samples exhibited bands in the expected absorption regions for proteins. The 5 wt.% gelatin gels without surfactant (NS) showed a characteristic band at 3343 cm^−1^ attributed to the presence of hydrogen bonds and amide-A. Moreover, the two bands at 1651 cm^−1^ and 1538 cm^−1^ corresponded to amide-I and amide-II, respectively. The 20 wt.% gelatin gel spectrum exhibited similar characteristics to the 5 wt.% gels with some changes in intensity and shift. Typical absorption bands were absorbed at 20 wt.% at 1653 cm^−1^ (amide-I) and 1539 cm^−1^ (amide-II).

SDS addition into the 5 and 20 wt.% gelatin gels led to shifts in the characteristic amide band positions due to gelatin–SDS physical interactions and micelles formation [46]. The frequency of the amide-I and amide-II bands for the 5 wt.% and 20 wt.% gelatin gels increased as the S/G ratio increased up to 0.075 and 0.0375, respectively, after which they started to decrease. This indicates a secondary structure development until those S/G ratios, followed by a structural decline. The characteristic amide-A band of the gelatin for both gelatin concentrations initially shifted to shorter wavenumbers, indicating gelatin–SDS interaction through increasing intermolecular hydrogel bonds. However, at the highest S/G ratio, 0.15, the amide-A band shifted to higher frequencies, indicating the proliferation of hydrophobic interactions. In addition, the peak range between the 2848 and 2960 cm^−1^ frequencies was assigned to the C–H stretching of alkane groups from the interaction of gelatin and SDS [47,48]. The asymmetric and symmetric CH_3_ stretching vibrational frequencies were located at 2957–2960 cm^−1^ and 2873–2879 cm^−1^, respectively. The asymmetric and symmetric CH_2_ stretching vibrational frequencies were found at 2926–2941 cm^−1^ and 2848–2857 cm^−1^, respectively. As expected, the CH_2_ stretching intensities were higher than those for the CH_3_, and both were more intense in samples containing SDS than in the pure gelatin ones.

CH_2_ stretching characteristics can be related to conformation and packing of the SDS molecules, because CH_2_ stretching is sensitive to methylene chains conformation, indicating a relatively ordered crystalline structure at CH_2_ symmetric stretching values lower than 2852 cm^−1^ [48]. The results showed that gelatin–SDS gels presented CH_2_ symmetric stretching bands between 2854 and 2857 cm^−1^, representing the presence of micelles and liquid crystals [48]. Another typical band associated with SDS presence is the sulfate asymmetric vibration band containing overlapping peaks, representing the conformational structure and the sulfate headgroup dipole components [48]. This SO_2_ asymmetric vibrational feature was found at 1235–1241 cm^−1^ and exhibited higher wavenumbers in the gelatin–SDS gels than in the pure gelatin gels.

### 2.4. Viscosity of the Solutions

**Figure 4:**
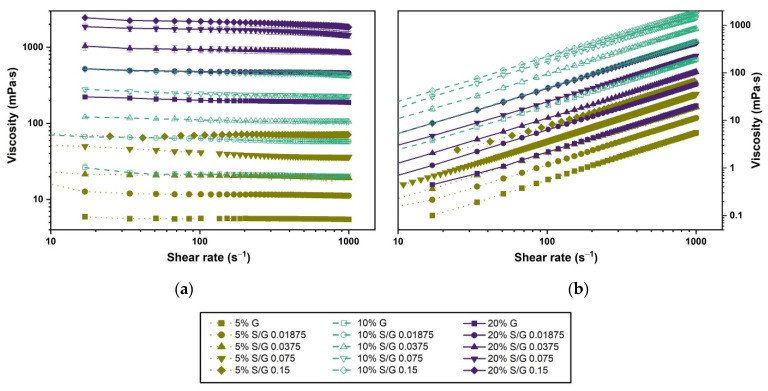
(**a**) Flow curves for gelatin and gelatin–SDS solutions at 40 °C. (**b**) Shear stress vs. shear rate curves for gelatin and gelatin–SDS solutions at 40 °C.

Figure 4a shows the flow curves for the gelatin and gelatin–SDS solutions. The lowest gelatin concentration solutions (5 wt.%) exhibited near-Newtonian behavior. However, increasing gelatin and SDS concentrations resulted in increasing shear-thinning at higher shear rates. This is attributable to the molecular entanglement associated with an increase in the molecular size of the gelatin solutions and an increasing number of gelatin–SDS complexes [38,49]. In addition, by extrapolation of the curves to zero shear rate, it can be seen that the yield strength of the solutions increased with an increase in gelatin and SDS concentrations.

**Figure 5:**
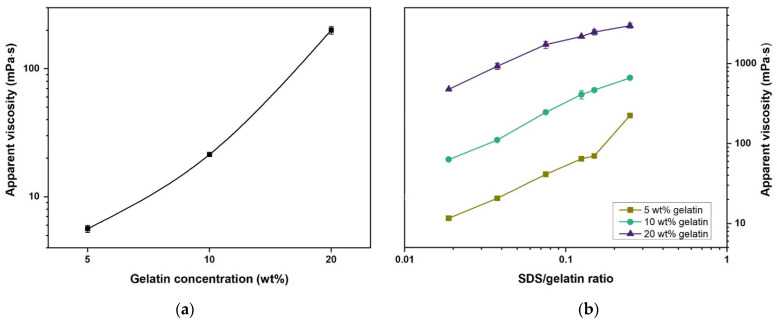
(**a**) Apparent viscosity of aqueous gelatin solutions as a function of gelatin concentration at 101.7 s^−1^ and 40 °C. (**b**) Apparent viscosity variation as a function of SDS/gelatin ratio for 5, 10, and 20 wt.% gelatin concentrations at 101.7 s^−1^ and 40 °C.

The gelatin–SDS interaction was manifested by an almost linear thickening of the solution, attributed to the gelatin molecules’ association and gelatin–SDS intramolecular and intermolecular interactions [13,38]. A gelatin concentration increase from 5 to 20 wt.% gave rise to a change in the solution’s viscosity from 6 to 200 mPa·s at a selected shear rate typically used for polymer blends mixing [50]. Thus, as typically found in polymer solutions, the viscosity of the samples increased with gelatin concentration (see Figure 5a). Surfactant incorporation into the gelatin solutions also gave rise to the solution’s thickening (see Figure 5b). The apparent viscosity was strongly dependent on the gelatin and SDS concentrations (see Figure 5), significantly affecting the hydrogels’ processing and functionality. Thus, the viscosity of the hydrogel solution must be properly considered during formulation and can be adjusted by the surfactant content in addition to the gelatin concentration and temperature. As an illustration, excessive viscosities may clog the dispensing needles in electrospinning or bioprinting and hinder gas introduction during mechanical foaming [9]. On the other hand, low viscosity can lead to the deformation of extruded gels, and it is also undesirable for hydrogel foaming, as a relatively high viscosity is required to arrest foam aging (i.e., drainage, coalescence, and coarsening) [9,51,52].

### 2.5. Rheological Study

The gelatin hydrogels’ gelation kinetics, gel strength, and gelling/melting points were investigated using the storage modulus (*G*’) and loss modulus (*G*”) generated under different test conditions. For materials showing viscoelastic behavior, such as gelatin, *G*’ and *G*” represent the material’s elastic and viscous behavior, respectively. The gel elasticity, *G*’, is directly related to the helix concentration and weak interactions between them [53]. In contrast, the viscous portion, *G*”, represents liquid-like behavior and generates from the internal friction between molecules and particles in the material, transforming deformation into frictional heat.

#### 2.5.1. Gelation Kinetics

**Figure 6:**
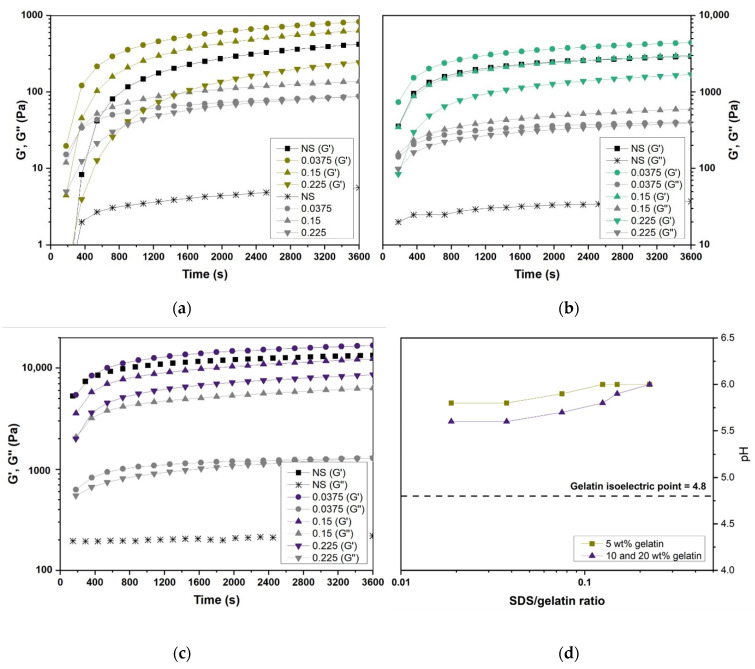
(**a**) *G*’-*G*’’ vs. time plot for gelatin and gelatin/SDS hydrogels at selected S/G ratios: (**a**) 5 wt.% gelatin concentration, (**b**) 10 wt.% gelatin concentration, (**c**) and 20 wt.% gelatin concentration. (**d**) pH variation in gelatin/SDS hydrogels solutions at 40 °C upon variation of S/G ratio.

The influence of the S/G ratio on gelation kinetics was investigated by isothermal (23 °C) time-dependent tests at constant deformation (f = 1 Hz, ɣ = 1%). Figure 6a–c shows *G*’ and *G*’’ as a function of time for hydrogels with varying S/G ratios at 5, 10, and 20 wt.% gelatin contents, respectively, and thus their transformation from a random coil state to a triple helix structure. As expected, the gelatin gels exhibited a viscoelastic behavior and the characteristic behavior of a gelling system. These can be divided into three stages: (1) an initial and sharp increase of the *G*’–*G*’’ curves, (2) the *G*’’-*G*’ crossover point (*G*’ = *G*’’), and (3) stabilization of the *G*’–*G*’’ curves, which both tend to plateau [54]. As gelation proceeded from a sol (liquid-like behavior predominance, *G*’’ > *G*’) to a gel (solid-like behavior predominance, *G*’ > *G*’’) state, *G*’ rapidly increased and surpassed *G*’’.

The black curves (NS *G*’ and NS *G*’’) in Figure 6a–c represent the gelatin gels’ storage and loss modulus with no SDS. As Ross-Murphy [55] observed, higher gelatin concentration solutions exhibited faster gelation kinetics and, in turn, shorter time to achieve the storage modulus plateau. Surfactant incorporation at 0.0375 S/G ratios accelerated the gelation kinetics compared to gelatin gels without surfactant for the three gelatin concentrations studied. An S/G ratio of 0.15 led to a slighter gelation kinetics acceleration for 5 wt.% gelatin gels but slightly decelerated 10 and 20 wt.% gelation. However, the three gelatin concentrations studied were slowed down when surfactant was incorporated at a 0.225 S/G ratio. In addition, 5 and 10 wt.% gelatin solutions did not gel for the timescale studied (60 min) when the S/G ratio was equal to or higher than 0.375 and 0.5, respectively. Thus, SDS behaved as a gelling accelerator of gelatin gels when incorporated at relatively low S/G ratios and as a gelatin denaturant when used at higher concentrations.

**Figure 7:**
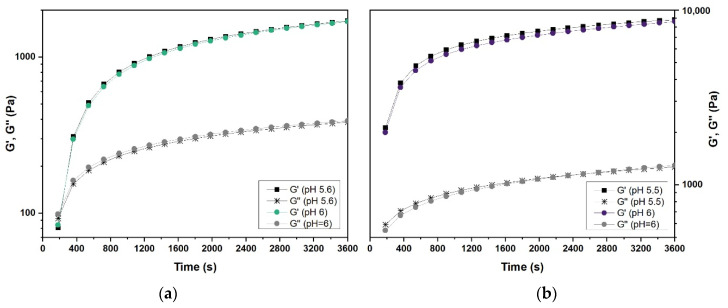
(**a**) Comparison of G’-G’’ vs. time plots of 10 wt.% hydrogels prepared at 0.225 S/G ratios at their natural pH (pH = 6) and pH modified to that of the 10 wt.% gelatin solutions without surfactant (pH = 5.6). (**b**) Comparison of G’-G’’ vs. time plots of 20 wt.% hydrogels prepared at 0.225 S/G ratios at their natural pH (pH = 6) and pH modified to that of the 20 wt.% gelatin solutions without surfactant (pH = 5.5).

Electrostatic interactions are expected to be predominant for charged molecules; thus, pH and ionic strength alterations in the aqueous phase are expected to affect the interactions substantially [38,39]. As can be seen in Figure 6d, the pH of the gelatin solutions slightly increased as SDS was incorporated. The pH of the gelatin gels at 5, 10, and 20 wt.% concentrations (without SDS) was 5.5–5.6 and increased to the same level of pH = 6 at an S/G ratio of 0.225. The pH rising as the S/G ratio increased (see Figure 6d) is attributed to surfactant molecules binding to the gelatin chain’s positive groups, increasing the solution’s negative charge. The increase in pH is expected to have a double effect on the solution, increasing gelatin coil expansion and decreasing the electrostatic surfactant binding [13]. Figure 7 shows that the slight pH increase when surfactant was added was not the main reason for gelation hindering at high S/G ratios. The pH correction of 0.225 S/G gels at 10 wt.% and 20 wt.% gelatin concentrations to the pH of the pure gelatin solutions showed little effect on the gelling behavior and storage modulus. However, more significant pH alterations are likely to affect gelation considerably. For further information on the influence of pH on the rheological properties of gelatin–surfactant systems, the work of Dreja et al. [22] is recommended. Therefore, it was considered unnecessary to compensate for the pH increase from the addition of SDS for gelation rate control purposes.

This section studied the gelation kinetics qualitatively; further work can be carried out by determining the gelation time by temperature-dependent rheological tests.

#### 2.5.2. Gel Strength

The strength of the gelatin and gelatin–SDS hydrogels was recorded as *G*’_60_, the storage modulus at 60 min of the isothermal (23 °C) time-dependent tests described above. Gel strength, *G*’_60_, was higher at higher gelatin concentrations (see Table 2), as this enhances crosslinking sites’ concentration and helix formation [56].

**Figure 8:**
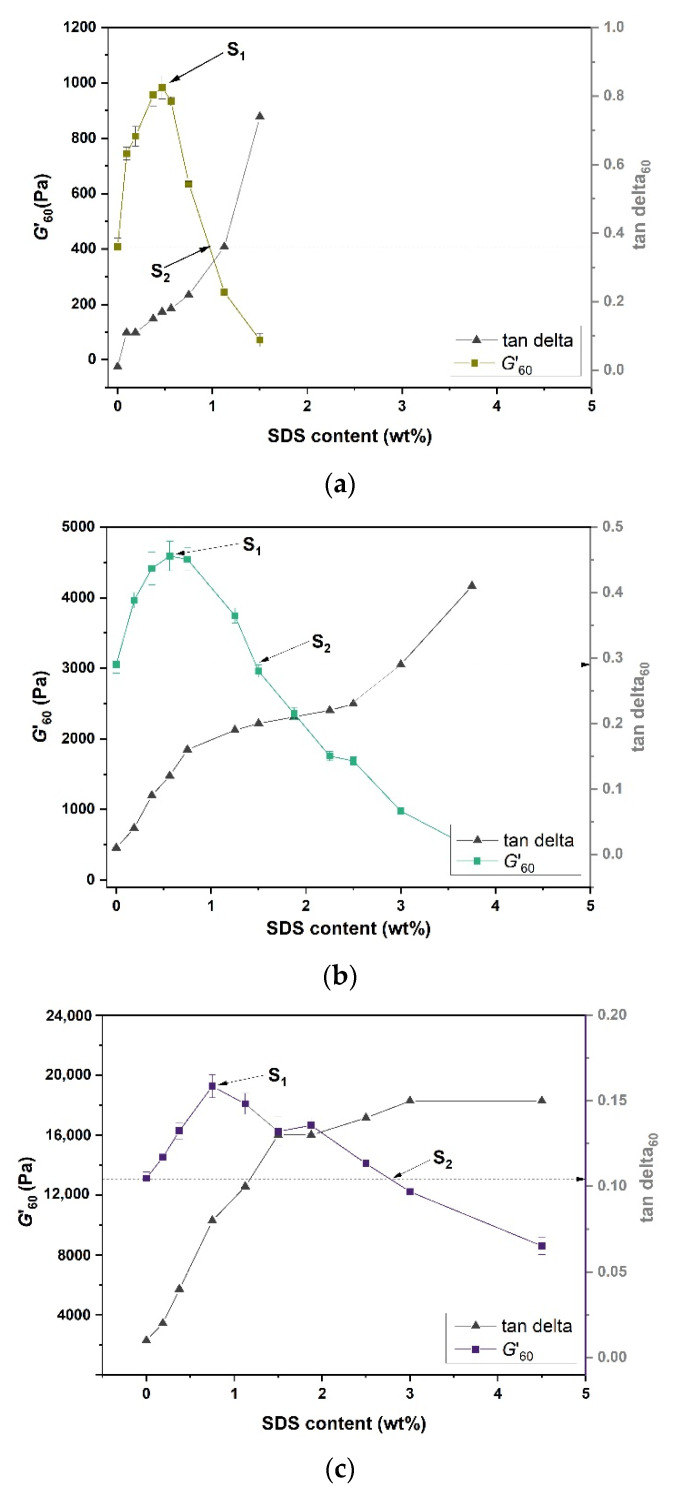
Equilibrium modulus (G’60) of gelatin and gelatin–SDS gels prepared after 1 h curing at 23 °C: (**a**) G’60 vs. SDS content for 5 wt.% gelatin concentration, (**b**) G’60 vs. SDS content for 10 wt.% gelatin concentration, and (**c**) G’60 vs. SDS content for 20 wt.% gelatin concentration.

Figure 8 compares the *G*’_60_ and tanδ_60_ of the gelatin and gelatin–SDS hydrogels at different SDS weight percentage contents. The amount of SDS added into the formulations considerably affected *G*’_60_. Two characteristic surfactant concentrations were identified: S_1_, corresponding to the surfactant concentration at which the G’_60_ of the gelatin–SDS hydrogels reached a maximum, and S_2_, corresponding to the surfactant concentration at which the G’_60_ of the gelatin–SDS hydrogels was equal to that of the pure gelatin gel (marked by the dotted lines), marking the onset of the gelatin–SDS hydrogel denaturation. Accordingly, three zones can be defined: (1) increase (where surfactant concentration, S, is lower than S_1_), (2) decline (where S_1_ < S < S_2_), and (3) collapse (where S > S_2_).

In the “increase” zone, G’_60_ initially rose as the SDS content increased up to S_1_. The maximum registered increase relative to that without SDS was most drastic for the 5 wt.% gelatin gels (about 2.5 times) and reduced to about 1.5 times for more concentrated gelatin gels at 10 and 20 wt.%, respectively. This maximum G’_60_ value was achieved at 0.09375 (0.46875 wt.%), 0.05625 (0.5625 wt.%), and 0.0375 (0.75 wt.%) S/G ratios for 5, 10, and 20 wt.% gelatin gels, respectively. Thus, the higher the gel strength (i.e., gelatin concentration), the lower the S/G ratio at which the maximum increase in elasticity was obtained. It has been argued that the gelatin–SDS interactions below S_1_ (the “increase” region) for gel strength are dominated by the overlapping of the gelatin chains with the surfactant micelles, forming crosslinks and favoring secondary structures by electrostatic and hydrophobic interactions [57]. These results were confirmed with FTIR results showing the development of such a secondary structure at its corresponding S/G ratios (see Table 2). The data suggested that SDS binding to gelatin chains led to complex formation, affecting intermolecular interactions and gelatin conformation due to the bands’ shifts in the amide bands’ positions. [18]. This behavior was also observed by Hirlekar et al. [25] in silk fiber–surfactant systems.

As the SDS concentration increased beyond S_1_ into the “decline” region, the gelatin–SDS interactions disrupted the triple helix formation, delaying and even preventing gelling at the timescale studied when the S/G ratio was equal to or higher than 0.375 and 0.5 for 5 wt.% and 10 wt.% gelatin concentrations, respectively (see Table 2). The hydrogels’ gel strength arises from the intermolecular bonds between adjacent helix units, so rigidity loss is attributed to an intermolecular bond loss favoring micelle–gelatin interactions [26]. FTIR and UV-Vis spectrophotometry results also suggested that in the presence of excessive SDS, the gels’ secondary structure was compromised by a reduction in the amide-I band wavenumber (see Table 1) and an increase in transmittance. At S_2,_ the benefit of gel strengthening by SDS was completely lost, and such a loss of gel strength was more significant for gels with lower gelatin contents. At the timescale studied, the G’–G’’ crossover did not take place for 5 wt.% and 10 wt.% gelatin hydrogels from 1.875 wt.% (0.375 S/G ratios) and 5 wt.% (0.5 S/G ratios) surfactant content, respectively, whereas such gelling disruption behavior was not observed in 20 wt.% gels at the studied S/G ratios. Ionic surfactants with alkyl chains, such as SDS, are known for acting as protein denaturants [29,36]. Protein denaturation involves the destruction of the secondary and tertiary structure of the protein molecules. The pearl-and-necklace model attributes denaturation to electrostatic repulsion between individual micelles, while the decorated micelle model assumes that protein more or less sequesters the micellar charges [12]. Nevertheless, as Otzen and Oliveberg [12] pointed out, it may be more instructive to acknowledge that protein–surfactant interactions are too diverse to be accommodated in one model. Thus, two main mechanisms of gelatin denaturation in the presence of SDS are proposed. The first is based on the competition between micellar binding and gelatin helix formation. The loss of intermolecular bonds due to micelle-rich linkages directly results from electrostatic interactions between the surfactant’s anionic and cationic groups in the gelatin’s backbone [26]. Secondly, denaturation results from a secondary role of the electrostatic binding and its influence on gelatin unfolding at high SDS concentrations. In addition, this accelerating unfolding and denaturing behavior is attributed to the elongated micelles forming at higher surfactant concentrations [58]. Unfolding leads to the exposure of the gelatin’s hydrophobic residues buried in the tertiary structure, which will end up interacting with the surfactant’s hydrophobic tails [59].

Figure 8 also presents tanδ_60_, the loss factor G”/G’ at 60 min, as a function of SDS concentration. Viscoelastic liquids are commonly classified by tanδ > 1, while viscoelastic solids are classified by tanδ < 1. During the isothermal time sweep test, tanδ decreased with time as gelation advanced, showing increased solid-like behavior as gelation proceeded, resulting in tanδ_60_ < 1 for gels at all SDS concentrations, which implied that the gels were viscoelastic solids. However, higher SDS concentration promoted the increasing viscous behavior of the hydrogels (i.e., higher tanδ_60_) due to protein unfolding, and this effect was more evident in gels with lower gelatin content (comparing tanδ_60_ in Figure 8a–c). The loss modulus, higher at higher tanδ_60_ values, represents dangling ends, free chains, and loops attached to the network. These are structural features that contribute to energy dissipation by friction rather than increasing elasticity [53].

#### 2.5.3. Gelling and Melting Temperatures

The gelling and melting temperatures of the 10 and 20 wt.% gelatin and gelatin–SDS hydrogels were determined by temperature ramp tests at different cooling and heating rates.

On heating from 23 °C to 40 °C, fully formed gelatin hydrogels exhibited a gel–sol transition. When gelatin hydrogels are in a gel state, *G*’ is higher than *G*’’. As the temperature increases, the gelatin hydrogel melts to form a gelatin solution. Both *G*’ and *G*” decrease, yet G’ decreases considerably more, resulting in a *G*’–*G*” crossover. This *G*’–G” crossover on heating indicates the material transition from a solid-dominant state to a liquid-dominant state, and it is commonly denominated as melting temperature (T_m_).

On cooling from 40 °C to 23 °C, the gelatin solutions underwent a sol–gel transition. At 40 °C, the gelatin solution is liquid-like (*G*” > *G*’). As the temperature decreases, the gelatin solutions transition to form a gelatin gel and both *G*’’ and *G*’ (and this one more considerably) increased and led to a *G*’’–*G*’ crossover on cooling, known as gelling temperature (T_g_).

Table 3 shows the gelling and melting temperatures of gelatin and gelatin–SDS gels at 10 wt.% and 20 wt.% at a fixed heating/cooling rate of 2 °C/min. Higher gelatin contents exhibited higher gelling and melting temperatures, as reported for gelatin systems [36,60,61]. As polymer concentration increases, the intermolecular helix associations are more likely to happen faster, leading to a more rapid increase in storage modulus and, thus, gelling temperature [62]. Furthermore, higher gelatin concentrations depict a higher number and more robust junction zones formed and, consequently, the energy required for melting [63]. However, a more substantial influence of gelatin content on gelling temperature than on melting temperature can be observed. The 10 wt.% gelatin solutions with and without SDS gelled at 21.91–26.12 °C and melted at 31.81–34.23 °C, while the 20 wt.% gelatin solutions with and without SDS gelled at 25.29–30.05 °C and melted at 33.92–35.19 °C. Thus, the hydrogels exhibited hysteresis between the gelling and melting temperatures. The gelling and melting temperature hysteresis was slightly higher at lower gelatin concentrations without surfactant (e.g., around 7.6 °C for the 10 wt.% gelatin compared to 5.5 °C for the 20 wt.% gelatin).

**Figure 9:**
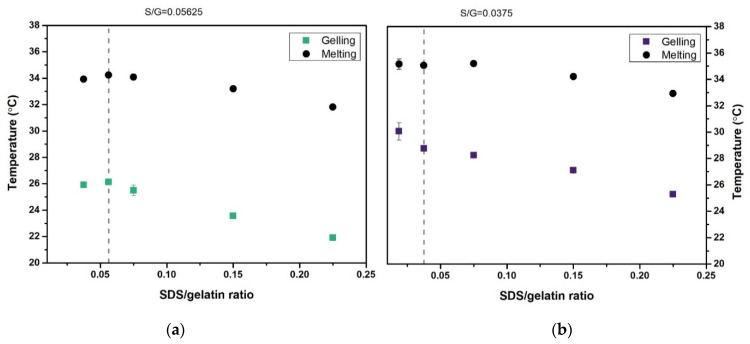
Melting and gelling temperatures of gelatin gels and solutions at different S/G ratios and 2 °C/min heating/cooling rate for (**a**) 10 wt.% gelatin concentrations and (**b**) 20 wt.% gelatin concentrations.

Both gelling and melting temperatures exhibited a similar trend to G’_60_ (see Figure 9). Gelling and melting temperatures increased to a maximum corresponding to SDS concentration for the maximum value of G’_60_, from which they decreased. The decline observed from the highest gelling/melting temperatures obtained with SDS addition (0.05621 and 0.375 S/G ratios for 10 and 20 wt.% gelatin gels, respectively) to the highest S/G ratio studied (0.225) was more significant for gelling (4.21 and 4.76 °C for 10 and 20 wt.% gelatin gels, respectively) than for melting temperatures (2.42 and 2.23 °C for 10 and 20 wt.% gelatin gels, respectively). Gelatin–SDS complexes needed higher energy for melting, attributed to higher triple helix stability and length [64], and thus an increase in melting temperature. Similarly, the increase in gelling temperature in the increased region is attributed to the gelatin chains overlapping with the surfactant micelles, forming micelles and accelerating gelling [57]. The decline observed for gelling/melting temperatures was more significant for gelling than melting temperatures due to higher SDS contents considerably hindering the intermolecular bonding of the gelatin chains, closely related to gelling.

**Figure 10:**
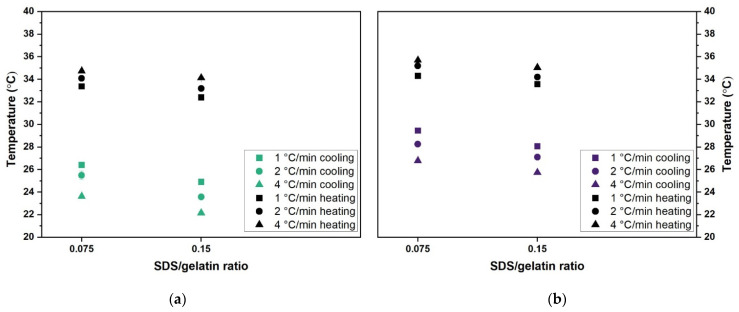
Melting and gelling temperatures of gelatin gels and solutions for 0.075 and 0.15 S/G ratios at 1, 2, and 4 °C/min heating/cooling rates for (**a**) 10 wt.% gelatin concentrations and (**b**) 20 wt.% gelatin concentrations.

Measurements of melting and gelling temperatures are known to be dependent on heating and cooling rates. Such a dependence is shown in Figure 10 by extending the heating/cooling rates studied to 1 °C/min and 4 °C/min. The same increase and decline trend for gelling/melting behavior was found for the selected S/G ratios at different cooling and heating rates (1 °C/min, 2 °C/min, and 4 °C/min). In addition, higher heating rates led to higher melting temperatures, while higher cooling rates led to a decrease in gelling temperature. Thus, this easy to tailor processing parameter can adjust the temperature-dependent rheological properties towards process optimization without altering formulation.

## 3. Conclusions

SDS is an effective surfactant for lowering the surface tension of aqueous solutions, but in the presence of highly surface-active polymers, such as gelatin, this functionality can be compromised. SDS can have additional roles in gelatin solutions and hydrogels, such as modifying the rheological properties, including viscosity, gelation kinetics, gel strength, and gelling/melting temperature.

Higher gelatin concentrations give rise to faster gelation, stronger hydrogels, and higher gelling/melting temperatures. In addition to gelatin concentration, the gelatin–SDS interaction also plays a significant role in the rheological properties. Lower SDS concentrations, below a certain optimum value, resulted in a beneficial increase in rheological properties (higher gel strength, faster gelation kinetics, and higher melting and gelling temperatures). In contrast, excessive SDS concentration beyond the optimum level caused a decline in the gelation kinetics, gel strength, *G*’_60_, and melting/gelling temperatures.

The results indicated an optimal SDS concentration (S_1_) relative to gelatin’s concentration at which the hydrogel’s strength is maximum and begins to decline up to an SDS concentration (S_2_), after which the positive effect of SDS addition ceased. This increase/decline in the gel’s strength trend can be correlated to the gelatin–SDS associations, which were highly dependent on the S/G ratio. The viscoelastic properties’ “increase” trend arises from the formation of gelatin–micelle crosslinks and the acceleration in the formation of secondary structures, while the decline is attributed to micellar binding competition with helix formation and gelatin tertiary structure unfolding.

The analysis of gelatin–SDS viscoelastic properties carried out here has extended our knowledge of the influence of anionic surfactants on the properties of high-strength gelatin hydrogels. The scope of this study was focused on the natural pH and ionic strength of the gelatin–SDS solutions, but it would be interesting to assess in detail the influence of these two parameters on the increase/decline curve for further work. In addition, for the accurate determination and analysis of gelation time of high-strength gelatin gels, temperature-dependent tests are recommended for future investigations.

## 4. Materials and Methods

### 4.1. Materials

240 Bloom type B gelatin produced from a mixture of cow and pig bones was purchased from Dongbao Bio-Tech Co Ltd. (Baotou, China). Bloom was measured using the method of the Gelatin Manufacturers Institute of America (GMIA) [65]. The gelatin’s average molecular weight (Mw), determined by gel permeation chromatography (GPC), was 122,400 g/mol. The moisture content of the as-received material was 11%, measured using a Mettler Toledo HE73 moisture analyzer (Columbus, OH, USA). The isoelectric point was 4.8, determined by zeta potential measurements at different pHs on a Zetasizer Nano-ZE apparatus (Malvern Instruments, UK) combined with a pH auto titrator. Sodium dodecyl sulfate (SDS), assay 98.5%, was supplied by BioFroxx (Einhausen, Germany). Deionized water was used to prepare all the solutions, and HCl, supplied by Dongguan Dongjiang Chemical Reagent Ltd. (Dongguan, China), was used as a buffer when pH adjustment was required.

### 4.2. Preparation of Gelatin–SDS Solutions

Aqueous gelatin–SDS solutions at 5 wt.%, 10 wt.%, and 20 wt.% gelatin concentrations were prepared by dissolving gelatin along with SDS at 60 °C with magnetic stirring at 250 rpm for 30 min. The gelatin–SDS solutions were allowed to stabilize at 40 °C for 20 min without stirring to ensure they were free from bubbles. In addition, when required, a small amount of HCl (up to 50 μL/100 mL) was incorporated dropwise for pH adjustment. This process was monitored using an Orion Star A221, portable pH meter (Thermo Fisher Scientific, Waltham, MA, USA).

### 4.3. Characterization

All the characterizations were carried out in triplicate.

#### 4.3.1. UV-Vis Spectroscopy

A LAMBDA 750s spectrophotometer (Perkin Elmer, Wellesley, MA, USA) equipped with a temperature-controlled Peltier device was used to assess the solutions’ solubility and the development of light-scattering aggregates. Transmittance measurements studied gelatin–SDS solutions at 40 °C at 600 nm. Samples were placed in a quartz cell with an optical path length of 10 mm.

#### 4.3.2. Surface Tension

The surface tension of the gelatin and gelatin–SDS solutions was measured at 40 °C by the Wilhelmy plate method using a K100 model tensiometer (Krüss, Hamburg, Germany). The surface tension of polymer/surfactant systems is in dynamic equilibrium; thus, a waiting time of 30 min before measurements was adopted to establish a relatively steady state.

#### 4.3.3. Fourier Transform Infrared (FTIR) Spectroscopy

FTIR absorption spectra of the gelatin and gelatin–SDS dry gels were recorded using a Frontier FT-IR Spectrometer (Perkin Elmer, Wellesley, MA, USA) at a resolution of 2 cm^−1^ with 32 scans over the wavenumber range of 400–4000 cm^−1^. The gels, in powder form, were mixed with KBr at a ratio of 1:100.

#### 4.3.4. Viscosity

The viscosity of the gelatin solutions at 40 °C was measured using a Haake Mars III rheometer (Thermo Fisher Scientific, Waltham, MA, USA) equipped with a Peltier temperature control system. The solutions were loaded into a 40 °C pre-heated double gap cup (3 mL capacity) and stabilized for 60 s. The measurements were carried out using an isothermal (40 °C) shear rate ramp test from 0.01 s^−1^ to 1000 s^−1^ with 60 measuring points in 600 s. The 10 s interval was chosen to minimize the start-up effects at lower shear rates (ɣ < 1 s^−1^).

#### 4.3.5. Rheology

Small amplitude oscillatory shear (SAOS) measurements were used to characterize the rheological properties of the gels. The rheological characterization was carried out using a Haake Mars III rheometer fitted with a Peltier temperature control system and 60 mm diameter parallel plate geometry. The bubble-free gelatin and gelatin–SDS solutions were dispensed on the pre-heated lower plate at 40 °C. The upper plate was lowered to the predetermined testing gap, and the excess solution was wiped off. A thin layer of silicone oil was applied around the edges to prevent solvent evaporation.

The linear viscoelastic region (LVR) was determined using the method by Zuidema et al. [66] with some modifications. After 5 min of conditioning at 40 °C, the liquid sample enclosed within a 1 mm gap was cooled to 23 °C and held for 60 min, which was found to be sufficient for achieving a gelation quasi-equilibrium in a time sweep at the three studied gelatin concentrations. Then, frequency and amplitude sweeps at 23 °C were conducted to identify appropriate testing ranges within the LVR. The storage *G*’ and loss *G*” moduli were recorded for these analyses, and the end of the linear region was considered a 10% deviation from the quasi-equilibrium plateau, which ended at 25.5% strain when the frequency (f) was 1 Hz for the most rigid hydrogel studied (20 wt.% gelatin, surfactant to gelatin ratio S/G = 0.0375). The parameters chosen for further characterization (isothermal time-dependent and temperature-dependent tests) had a 1 Hz frequency, 1% strain, and 1 mm gap unless stated otherwise.

- Isothermal time-dependent rheological behavior: The isothermal time-dependent rheological behavior of the gelatin and gelatin–SDS solutions was studied to assess gelation kinetics and the storage modulus. After 5 min of stabilization at 40 °C (t = 0 min), the hydrogel sample was cooled and held at 23 °C under isothermal conditions during a 60 min-time sweep. The storage and loss modulus variation with time and the storage modulus value at t = 60 min (*G*’_60_) were recorded. In addition, a comparison of the time-dependent rheological behavior between gelatin–SDS solutions at 0.225 S/G ratios at its natural pH and those buffered to the pH of the pure gelatin solution was made to assess the influence of the pH variation upon SDS incorporation.
- Temperature-dependent rheological behavior: The temperature-dependent viscoelastic behavior of 10 wt.% and 20 wt.% gelatin hydrogels at different SDS content was investigated to determine the gelling and melting temperatures. For gelling temperature determination, after 5 min of conditioning at 40 °C, the gelatin solutions were allowed to cool from 40 °C to 20 °C at controlled rates of 1 °C/min, 2 °C/min, and 4 °C/min, respectively, at a constant frequency, 1 Hz, shear strain, ɣ = 3–6%, and 0.5 mm gap. The adjustments in strain and the gap setting were necessary to enhance the torque signals and the accuracy of the *G*’ and *G*” moduli. For melting temperature determination, the hydrogels were allowed to stabilize for 1000 s (time at which all the hydrogels’ storage modulus had entered the G’ plateau) at 23 °C and were then heated to 40 °C at controlled rates of 1 °C/min, 2 °C/min, and 4 °C/min, respectively, at the same testing conditions mentioned for gelling temperature determination (frequency = 1 Hz, ɣ = 3–6%, and 0.5 mm gap).

## Figures and Tables

**Table 1:** Main infrared absorption band assignments for gelatin and gelatin–SDS dry gels prepared at 5 and 20 wt.% at different S/G ratios.

IR Band Assignment	Wavenumber (cm^−1^)									
	5 wt.% Gelatin S/G					20 wt.% Gelatin S/G				
	NS ^1^	0.019	0.0375	0.075	0.15	NS ^1^	0.019	0.0375	0.075	0.15
Amide-A	3343	3317	3317	3294	3314	3381	3342	3330	3329	3365
Amide-B	3073	3080	3080	3079	3081	3072	3077	3078	3078	3077
Amide-I	1651	1660	1660	1666	1662	1653	1663	1663	1664	1648
Amide-II	1538	1551	1552	1547	1545	1539	1543	1546	1541	1540
Asymmetric CH_3_ stretching	2959	2959	2960	2957	2957	2958	2958	2958	2957	2957
Symmetric CH_3_ strecthing	2878	2879	2878	2878	2878	2877	2877	2877	2878	2873
Asymmetric CH_2_ stretching	2941	2938	2936	2938	2929	2931	2931	2933	2931	2926
Symmetric CH_2_ stretching	2848	2856	2856	2855	2854	2848	2855	2855	2857	2856
Asymmetric SO_2_ vibration	1235	1241	1241	1240	1237	1236	1239	1238	1237	1237

^1^ NS: No Surfactant.

**Table 2:** Gel strength *G*’_60_ and tanδ_60_ for gelatin gels at 5 wt.%, 10 wt.%, and 20 wt.% gelatin contents at different S/G ratios.

S/G Ratio	5 wt.% Gelatin			10 wt.% Gelatin			20 wt.% Gelatin		
	wt.%	*G’*_60_ (Pa)	tanδ_60_	wt.%	*G’*_60_ (Pa)	tanδ_60_	wt.%	*G’*_60_ (Pa)	tanδ_60_
-	0	407 ± 32	0.01	0	3054 ± 124	0.01	0	13,117 ± 410	0.01
0.009375	0.04688	-	-	0.09375	-	-	0.1875	14,510 ± 42	0.02
0.01875	0.09375	744 ± 23	0.11	0.1875	3963 ± 111	0.04	0.375	16,281 ± 549	0.04
0.0375	0.1875	807 ± 36	0.11	0.375	4413 ± 232	0.09	0.75	19,262 ± 329	0.08
0.05625	0.28125	-	-	0.5625	4590 ± 208	0.12	1.125	18,088 ± 764	0.1
0.075	0.375	957 ± 42	0.15	0.75	4544 ± 162	0.16	1.5	16,245 ± 700	0.13
0.09375	0.46875	984 ± 42	0.17	0.9375	-	-	1.875	16,645 ± 552	0.13
0.1125	0.5625	933 ± 16	0.18	1.125	-	-	2.25	-	-
0.125	0.625	-	-	1.25	3745 ± 106	0.19	2.5	14,094 ± 205	0.14
0.15	0.75	635 ± 8	0.22	1.5	2960 ± 86	0.2	3	12,216 ± 167	0.15
0.1875	0.9375	-	-	1.875	2358 ± 86	0.21	3.75	-	-
0.225	1.125	244 ± 7	0.36	2.25	1757 ± 86	0.22	4.5	8602 ± 7	0.15
0.25	1.25	-	-	2.5	1688 ± 11	0.23	5	-	-
0.3	1.5	71 ± 24	0.74	3	978 ± 14	0.29	6	-	-
0.375	1.875	No *G’’*-*G’* cross-over		3.75	449 ± 1	0.41	7.5	-	-
0.5	2.5			5	No *G*’’-*G*’ crossover		10	-	-

**Table 3:** Gelling (T_g_) and melting temperature (T_m_) of gelatin and gelatin–SDS gels at 10 wt.% and 20 wt.% gelatin concentrations.

S/G Ratio	10 wt.% Gelatin			20 wt.% Gelatin		
	SDS (wt.%)	T_g_ (°C)	T_m_ (°C)	SDS (wt.%)	T_g_ (°C)	T_m_ (°C)
-	0	25.13 ± 0.17	32.73 ± 0.12	0	28.92 ± 0.15	34.39 ± 0.01
0.01875	0.188	-	-	0.375	30.05 ± 0.66	35.15 ± 0.39
0.0375	0.375	25.91 ± 0.17	33.92 ± 0.17	0.75	28.76 ± 0.02	35.05 ± 0.01
0.05625	0.056	26.12 ± 0.21	34.23 ± 0.19	1.125	-	-
0.075	0.75	25.50 ± 0.39	34.08 ± 0.05	1.5	28.25 ± 0.11	35.19 ± 0.20
0.15	1.5	23.60 ± 0.04	33.19 ± 0.10	3	27.10 ± 0.07	34.20 ± 0.02
0.225	2.25	21.91 ± 0.08	31.81 ± 0.07	4.5	25.29 ± 0.03	32.92 ± 0.05

## Data Availability

Derived data supporting the findings of this study are available from the corresponding author (D.W.) upon request.

## References

[1] Pourjabbar B., Biazar E., Heidari Keshel S., Ahani-Nahayati M., Baradaran-Rafii A., Roozafzoon R., Alemzadeh-Ansari M.H. (2021). Bio-polymeric hydrogels for regeneration of corneal epithelial tissue. Int. J. Polym. Mater. Polym. Biomater..

[2] Ahsan A., Tian W.-X., Farooq M.A., Khan D.H. (2021). An overview of hydrogels and their role in transdermal drug delivery. Int. J. Polym. Mater. Polym. Biomater..

[3] Batista R.A., Espitia P.J.P., Quintans J.D.S.S., Freitas M.M., Cerqueira M.Â., Teixeira J.A., Cardoso J.C. (2019). Hydrogel as an alternative structure for food packaging systems. Carbohydr. Polym..

[4] Jaipan P., Nguyen A., Narayan R.J. (2017). Gelatin-based hydrogels for biomedical applications. MRS Commun..

[5] Nur Hanani Z.A., Roos Y.H., Kerry J.P. (2014). Use and application of gelatin as potential biodegradable packaging materials for food products. Int. J. Biol. Macromol..

[6] Baumgartner M., Hartmann F., Drack M., Preninger D., Wirthl D., Gerstmayr R., Lehner L., Mao G., Pruckner R., Demchyshyn S. (2020). Resilient yet entirely degradable gelatin-based biogels for soft robots and electronics. Nat. Mater..

[7] Rodríguez-Rodríguez R., Espinosa-Andrews H., Velasquillo-Martínez C., García-Carvajal Z.Y. (2020). Composite hydrogels based on gelatin, chitosan and polyvinyl alcohol to biomedical applications: A review. Int. J. Polym. Mater. Polym. Biomater..

[8] Torres-García R., Flores-Estrada J., Cauich-Rodríguez J.V., Flores-Reyes M., Flores-Merino M. (2020). V Design of a polyacrylamide and gelatin hydrogel as a synthetic extracellular matrix. Int. J. Polym. Mater. Polym. Biomater..

[9] You F., Wu X., Chen X. (2017). 3D printing of porous alginate/gelatin hydrogel scaffolds and their mechanical property characterization. Int. J. Polym. Mater. Polym. Biomater..

[10] Poursamar S.A., Hatami J., Lehner A.N., Da Silva C.L., Ferreira F.C., Antunes A.P.M. (2016). Potential application of gelatin scaffolds prepared through in situ gas foaming in skin tissue engineering. Int. J. Polym. Mater. Polym. Biomater..

[11] Sonawane R.O., Patil S.D. (2017). Gelatin–κ-carrageenan polyelectrolyte complex hydrogel compositions for the design and development of extended-release pellets. Int. J. Polym. Mater. Polym. Biomater..

[12] Otzen D. (2011). Protein-surfactant interactions: A tale of many states. Biochim. Biophys. Acta-Proteins Proteom..

[13] Howe A.M., Wilkins A.G., Goodwin J.W. (1992). The Interactions Between Gelatin and Surfactants—A Rheological Study. J. Photogr. Sci..

[14] Touhami Y., Rana D., Neale G., Hornof V. (2001). Study of Polymer–Surfactant Interactions via Surface Tension Measurements. Colloid Polym. Sci..

[15] Derkach S.R. (2015). Interfacial layers of complex-forming ionic surfactants with gelatin. Adv. Colloid Interface Sci..

[16] Misra P.K., Meher J., Maharana S. (2016). Investigation on the gelatin-surfactant interaction and physiochemical characteristics of the mixture. J. Mol. Liq..

[17] Dias S.V.E., Züge L.C.B., Santos A.F., Scheer A.D.P. (2018). Effect of surfactants and gelatin on the stability, rheology, and encapsulation efficiency of W1/O/W2 multiple emulsions containing avocado oil. J. Food Process Eng..

[18] Deng L., Kang X., Liu Y., Feng F., Zhang H. (2017). Effects of surfactants on the formation of gelatin nanofibres for controlled release of curcumin. Food Chem..

[19] Lin L.-H., Chen K.-M. (2006). Preparation and surface activity of gelatin derivative surfactants. Colloids Surf. A Physicochem. Eng. Asp..

[20] Banerjee S., Bhattacharya S. (2012). Food Gels: Gelling Process and New Applications. Crit. Rev. Food Sci. Nutr..

[21] Rao A., Kim J., Thomas R.R. (2005). Interfacial rheological studies of gelatin-sodium dodecyl sulfate complexes adsorbed at the air-water interface. Langmuir.

[22] Dreja M., Heine K., Tieke B., Junkers G. (1997). Effects of functionalized latex particles and anionic surfactants on the flow behavior of aqueous gelatin dispersions. J. Colloid Interface Sci..

[23] Wu X., Hou J., Li M., Wang J., Kaplan D.L., Lu S. (2012). Sodium dodecyl sulfate-induced rapid gelation of silk fibroin. Acta Biomater..

[24] Sovilj V., Milanovic J., Petrovic L. (2013). Influence of gelatin-sodium stearoyl lactylate interaction on the rheological properties of gelatin gels. Colloids Surf. A Physicochem. Eng. Asp..

[25] Hirlekar S., Ray D., Aswal V.K., Prabhune A., Nisal A., Ravindranathan S. (2019). Silk Fibroin-Sodium Dodecyl Sulfate Gelation: Molecular, Structural, and Rheological Insights. Langmuir.

[26] Abed M.A., Bohidar H.B. (2005). Surfactant induced softening in gelatin hydrogels. Eur. Polym. J..

[27] Griffiths P.C., Cheung A.Y.F. (2002). Interaction between surfactants and gelatin in aqueous solutions. Mater. Sci. Technol..

[28] Henriquez M., Abuin E., Lissi E. (1993). Interactions of ionic surfactants with gelatin in fluid solutions and the gel state studied by fluorescence techniques, potentiometry, and measurements of viscosity and gel strength. Colloid Polym. Sci..

[29] Ao M., Xu G., Kang W., Meng L., Gong H., Zhou T. (2011). Surface rheological behavior of gelatin/ionic liquid-type imidazolium gemini surfactant mixed systems. Soft Matter.

[30] Šišáková M., Asaumi Y., Uda M., Seike M., Oyama K., Higashimoto S., Hirai T., Nakamura Y., Fujii S. (2020). Dodecyl sulfate-doped polypyrrole derivative grains as a light-responsive liquid marble stabilizer. Polym. J..

[31] Clar J.G., Silvera Batista C.A., Youn S., Bonzongo J.-C.J., Ziegler K.J. (2013). Interactive Forces between Sodium Dodecyl Sulfate-Suspended Single-Walled Carbon Nanotubes and Agarose Gels. J. Am. Chem. Soc..

[32] Yang R., Okonkwo O.S., Zurakowski D., Kohane D.S. (2018). Synergy between chemical permeation enhancers and drug permeation across the tympanic membrane. J. Control. Release.

[33] Knox W.J., Parshall T.O. (1970). The interaction of sodium dodecyl sulfate with gelatin. J. Colloid Interface Sci..

[34] Onesippe C., Lagerge S. (2009). Study of the complex formation between sodium dodecyl sulphate and gelatin. Colloids. Surf. A Physicochem. Eng. Asp..

[35] Buron C., Filiatre C., Membrey F., Foissy A., Argillier J.F. (2004). Interactions between gelatin and sodium dodecyl sulphate: Binding isotherm and solution properties. Colloid. Polym. Sci..

[36] Ortiz-Zarama M.A., Camacho-Diaz B.H., Jiménez-Aparicio A.R., Solorza-Feria J. (2017). Effect of sodium dodecyl sulfate on the physical properties of gelatin/multi-walled carbon nanotubes solutions and films. Rev. Mex. Ing. Quim..

[37] Kronberg B., Holmberg K., Lindman B. (2014). Surface Chemistry of Surfactants and Polymers.

[38] Greener J., Contestable B.A., Bale M.D. (1987). Interaction of Anionic Surfactants with Gelatin: Viscosity Effects. Macromolecules.

[39] Malik W.U., Ashraf S.M. (1970). Viscometric studies on the interaction of sodium dodecyl sulphate with transfusion gelatin. Kolloid-Z. Und Z. Für Polym..

[40] Kronberg B., Holmberg K., Lindman B. (2014). Types of surfactants, their synthesis, and applications. Surf. Chem. Surfactants Polym..

[41] George J., Sudheesh P., Reddy P.N., Sreejith L. (2010). Studies on the interaction of SDS with gelatin in presence of urea derivatives. J. Dispers. Sci. Technol..

[42] ToolBox E. Surface Tension. https://www.engineeringtoolbox.com/surface-tension-d_962.html.

[43] Umlong I.M., Ismail K. (2007). Micellization behaviour of sodium dodecyl sulfate in different electrolyte media. Colloids Surf. A Physicochem. Eng. Asp..

[44] Kong J., Yu S. (2007). Fourier Transform Infrared Spectroscopic Analysis of Protein Secondary Structures. Acta Biochim. Biophys. Sin..

[45] Hoque M.S., Benjakul S., Prodpran T. (2010). Effect of heat treatment of film-forming solution on the properties of film from cuttlefish (Sepia pharaonis) skin gelatin. J. Food Eng..

[46] Kamyar S., Ahmad M., Zin W., Yunus W., Ibrahim N., Jokar M., Darroudi M. (2010). Synthesis and Characterization of Silver/Polylactide Nanocomposites. World Acad. Sci. Eng. Technol..

[47] Kanmani P., Rhim J.-W. (2014). Physical, mechanical and antimicrobial properties of gelatin based active nanocomposite films containing AgNPs and nanoclay. Food Hydrocoll..

[48] Viana R., Silva A., Pimentel A. (2012). Infrared Spectroscopy of Anionic, Cationic, and Zwitterionic Surfactants. Adv. Phys. Chem..

[49] Ludmila A., Dyshlyuk L. (2016). Study of viscosity of aqueous solutions of natural polysaccharides. Sci. Evol..

[50] Mezger T.G. (2014). The Rheology Handbook.

[51] Songchotikunpan P., Tattiyakul J., Supaphol P. (2008). Extraction and electrospinning of gelatin from fish skin. Int. J. Biol. Macromol..

[52] Landrock A.H., Landrock A.H. (1995). Handbook of Plastic Foams: Types, Properties, Manufacture and Applications.

[53] Joly-Duhamel C., Hellio D., Ajdari A., Djabourov M. (2002). All gelatin networks: 2. The master curve for elasticity. Langmuir.

[54] Fonkwe L.G., Narsimhan G., Cha A.S. (2003). Characterization of gelation time and texture of gelatin and gelatin-polysaccharide mixed gels. Food Hydrocoll..

[55] Ross-Murphy S.B. (1997). Structure and rheology of gelatin gels. Imaging Sci. J..

[56] Joly-Duhamel C., Hellio D., Djabourov M. (2002). All gelatin networks: 1. Biodiversity and physical chemistry. Langmuir.

[57] Bhakta A., Ruckenstein E. (1997). Decay of standing foams: Drainage, coalescence and collapse. Adv. Colloid Interface Sci..

[58] Clint J.H. (1992). Surfactant Aggregation.

[59] Magdassi S., Kamyshny A., Magdassi S. (1996). Surface Activity of Proteins: Chemical and Physicochemical Modifications.

[60] Osorio F.A., Bilbao E., Bustos R., Alvarez F. (2007). Effects of concentration, bloom degree, and pH on gelatin melting and gelling temperatures using small amplitude oscillatory rheology. Int. J. Food Prop..

[61] Pang Z., Deeth H., Sopade P., Sharma R., Bansal N. (2014). Rheology, texture and microstructure of gelatin gels with and without milk proteins. Food Hydrocoll..

[62] Van Den Bulcke A.I., Bogdanov B., De Rooze N., Schacht E.H., Cornelissen M., Berghmans H. (2000). Structural and rheological properties of methacrylamide modified gelatin hydrogels. Biomacromolecules.

[63] Haug I.J., Draget K.I., Smidsrød O. (2004). Physical and rheological properties of fish gelatin compared to mammalian gelatin. Food Hydrocoll..

[64] Gornall J.L., Terentjev E.M. (2008). Helix-coil transition of gelatin: Helical morphology and stability. Soft Matter.

[65] GMIA (2012). Gelatin Handbook. https://nitta-gelatin.com/wp-content/uploads/2018/02/GMIA_Gelatin-Handbook.pdf.

[66] Zuidema J.M., Rivet C.J., Gilbert R.J., Morrison F.A. (2014). A protocol for rheological characterization of hydrogels for tissue engineering strategies. J. Biomed. Mater. Res.—Part B Appl. Biomater..

